# Timing is everything: A connection between medulloblastoma prognosis and foetal cerebellar development

**DOI:** 10.1111/nan.12903

**Published:** 2023-05-06

**Authors:** Daniel Williamson, Edward C. Schwalbe, Simon Bailey, Steven C. Clifford

**Affiliations:** ^1^ Wolfson Childhood Cancer Research Centre, Translational and Clinical Research Institute, Newcastle University Centre for Cancer Newcastle University Newcastle upon Tyne UK; ^2^ Department of Applied Sciences Northumbria University Newcastle upon Tyne UK

**Keywords:** cerebellar, development, medulloblastoma, scRNA‐seq, transcriptomics

## Abstract

The childhood brain tumour medulloblastoma is typically classified into multiple discrete molecular subgroups with characteristic DNA methylation and expression patterns. Several of these subgroups are used as, or proposed to be, an effective basis for treatment stratification. Here, we highlight the close connection between the findings described in a recent series of studies which, together, strongly imply a continuous association between survival outcome, the transcriptional profile of a Group3/Group4 (i.e. non‐WNT/non‐SHH) medulloblastoma and the specific point during early foetal cerebellar development at which initial pathogenic disruption took place. This has important implications for future efforts to model the disease by incorporating driving molecular features into their specific developmental context. This further suggests that instead of relying upon discrete DNA methylation subgroups, using expression biomarkers as the basis of a continuous risk predictor may produce a more effective risk stratification of patients with Group3/Group4 medulloblastoma.

## Graphical abstract

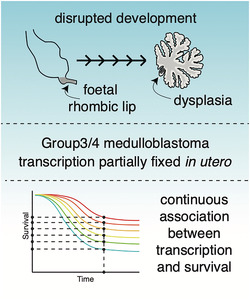

**Key points**

- Recent studies describe a continuous association between survival outcome and expression profile in Group3/Group4 medulloblastoma.
- Tumour expression profile is related to disruption at specific points in early foetal cerebellar development.
- Expression biomarkers may be used as the basis of a continuous risk predictor for effective risk stratification.

Molecular‐biological prognostication in medulloblastoma has been driven, over the last decade, by the discovery of increasing numbers of molecular subgroups and their associated mutations. Initially, medulloblastoma was divided by transcriptional profiling into SHH, WNT, Group3 (MB_Grp3_) and Group4 (MB_Grp4_) [1]; each has now been further subdivided into subgroups by DNA methylation patterns [2, 3, 4]. For example, the combined Group3/Group4 medulloblastoma (MB_Grp3/Grp4_) was divided into eight further subgroups (I–VIII) [5]. While of great biological interest, this atomisation of an already rare disease presents a practical problem to clinical trialists aiming to preserve statistical power. Here, we highlight the close connections between the findings described in a series of papers published in close succession by ourselves and others [6, 7, 8, 9], which together support the prognostic potential of gene expression across MB_Grp3/Grp4_ medulloblastoma as a whole and its relationship to normal cerebellar development.

Our recent study [9] analysed transcriptional profiles—in MB_Grp3/Grp4_ combined—to describe a single transcriptional continuum, a pattern of continuous expression changes that links all MB_Grp3_ and MB_Grp4_ patients. We devised a ‘G3/G4’ score to represent this expression pattern and used this score to place each patient at a unique position along the continuum between two extremes (i.e., the archetypal MB_Grp3_ and MB_Grp4_ transcriptional states). Position on the continuum was reflective of an individual's clinicopathology and significantly related to 5‐year survival.

Korshunov et al. [7] also recently performed a transcriptomic analysis of MB_Grp3_, describing differences in gene expression between patients who died vs those who survived 5 years post diagnosis. They identified six differentially expressed genes which were associated with high‐risk disease—*MYC*, *KIRREL2*, *ITPRIPL1*, *DCAF4*, *NPW* and *CDT1*—highlighting *KIRREL2* expression in particular, as prognostic, independent of other clinico‐molecular features, most notably MB_Grp3/Grp4_ DNA methylation subgroups (I–VIII) [5]. High *KIRREL2* expression was present in all MB_Grp3_ subgroups and, in combination with other risk factors, stratified disease risk with good accuracy.

While Korshunov et al. [7] divided their patients into high and low expressors, the survival association we described was linear and continuous; the higher an individual's G3/G4 score (i.e., the more ‘MB_Grp3_‐like’), the worse the prognosis. Notably, within our MB_Grp3/Grp4_ cohort, the expression of *KIRREL2* is log‐linearly correlated to the G3/G4 score and a major contributor (22nd out of 56,546 transcripts) to the MB_Grp3/Grp4_ continuum signature (Figure 1A); indeed, each of the top 6 genes described are significantly correlated with the G3/G4 score and with survival (Figure 1B–D). This strongly suggests that the results described by Korshunov et al. [7] are substantially part of the phenomenon described by the G3/G4 continuum.

**FIGURE 1:**
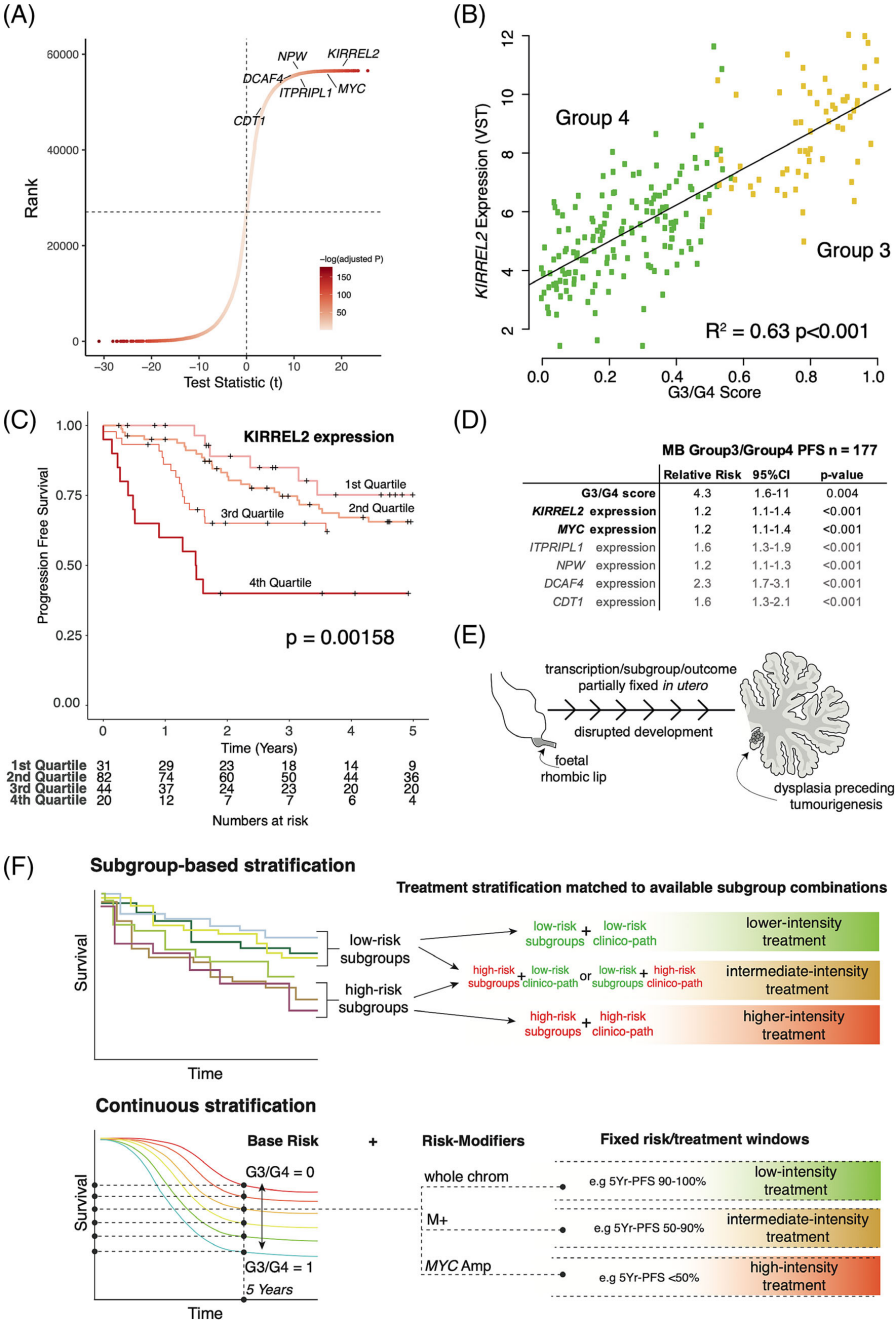
(A) Scatterplot showing all genes ordered by rank and correlation (*t*‐test statistic) with the G3/G4 score, that is, the transcriptional continuum. Colour represents a BH‐adjusted *p* value. All six genes highlighted by Korshunov et al. rank highly and are significantly positively correlated with *KIRREL2* ranking the highest. (B) Scatterplot showing significant log‐linear correlation (*p* < 0.001) between *KIRREL2* expression (VST = variance stabilised transform on a log scale) and G3/G4 score (continuum). Log‐linear line of best fit is shown. (C) Kaplan–Meier plot showing significant differences (log‐rank test for trend) in MB_Grp3/Grp4_ progression‐free survival by *KIRREL2* expression (divided into four quartiles). (D) Table of univariable Cox regression results showing relative risk (RR) of an event (progression/relapse or death) in MB_Grp3/Grp4_ as a continuous function of G3/G4 score or expression of six genes highlighted by Korshunov et al. Note that RR for a continuous score is RR per unit of measurement and that the range of each variable is different, that is, G3/G4 score scaled between 0 and 1 hence the higher RR. (E) Mini‐schema highlighting that transcription, subtype and risk of death are in large part fixed *in utero* prior to full transformation/tumourigenesis under the model proposed by Hendrikse et al. (F) Conceptual schema highlighting two alternative approaches to assigning patients to treatment stratifications. ‘Subtype‐based’ stratification—largely the approach pursued at present—attempting to agglomerate DNA methylation subtypes into groups with similar average outcomes and subsequently to find relevant clinico‐pathological characteristics that in combination place individuals into stratification windows (higher/intermediate/lower intensity) equate to a given average risk for the agglomerated groups. ‘Continuous Stratification,’ an alternative, perhaps more in keeping with the molecular biology, estimates a base level of 5‐year risk as a continuous value derived from the transcriptional continuum (G3/G4 score), afterwards applying any independent risk factors as modifiers to the base risk (examples given, M+ = presence of metastases, whole chrom = whole chromosome aberration phenotype described by Goschzik et al., *MYC* amp = *MYC* amplification). In this model, the treatment stratifications are fixed according to predefined risk windows. An individual's predicted 5‐year risk rather than specifically their DNA methylation subtype is the key factor for treatment stratification.

Our G3/G4 expression continuum—of which *KIRREL2* expression is a key constituent—was also projected onto an scRNA‐seq expression atlas of early human foetal cerebellar development [10], showing that the continuum is mirrored by a specific developmental trajectory—beginning with early rhombic lip (RL) precursors (most MB_Grp3_‐like) and ending with differentiated unipolar brush cells (UBCs) (most MB_Grp4_‐like)—linking individual tumours to specific developmental states by their position on the continuum. *KIRREL2* is a gene involved in cerebellar development, usually regarded as an early GABAergic cell fate determinant. This is perhaps counter‐intuitive for an RL/UBC (primarily glutamatergic) derived tumour, although recent descriptions of a multipotent posterior transitory zone between the ventricular zone and RL border may provide some explanation [11].

Smith et al. [8] and Hendrikse et al. [6] also recently published similar findings, that is, a single developmental RL/UBC lineage as the origin of the MB_Grp3/Grp4_ subgroups based on analysis of the same scRNA‐seq atlas [12]. Both studies aligned similar G3/G4 transcriptional patterns to two spatially distinct ventricular and subventricular compartments within early human RL development. Hendrikse et al. [6] further explored how the development of cells might be interrupted by disruption of the CBFA complex. They proposed the existence of persistent rhombic lip (PeRL), a postulated premalignant dysplasia within the cerebellar nodulus, resulting from disrupted foetal development/differentiation. They conflated PeRLs with previous reports of nodulus hyperplasia/genetic alterations dating back to the 1960s [13] and 1990s [14], surmising that further genetic hits could result in medulloblastoma.

Taken together, this series of recent studies forms a throughline suggesting that timing of initial disruption of early foetal development lineages fixes aspects of their tumour transcriptional profile at a given point and that this tracks closely to an individual's chance of surviving following treatment (Figure 1E).

If a more ‘MB_Grp3_‐like’ position upon the continuum, exemplified by high levels of *KIRREL2* expression, denotes an ‘earlier’ developmental disruption, why should those tumours be more aggressive and difficult to treat? It could be by virtue of their age at diagnosis—position on the continuum tracks closely to the average age of diagnosis [9]—although when controlling for age a significantly worse prognosis is still observed. Is there something intrinsic to the retained developmental biology which creates a more aggressive or treatment‐resistant cell? We may speculate about the expression of *MYC* or more broadly about an undifferentiated/proliferative phenotype, but it is not yet obvious specifically why they should be more resistant to therapy.

If a disruption occurs during foetal development, there is a substantial latent period, but why should premalignancies at one end of the continuum/developmental lineage lie dormant for 3–4 years on average and at the other end, for several years more? It does not appear that medulloblastomas occurring in older patients (e.g. subtype VIII) require time to accrue more mutations or that the mutations required are—by virtue of the mode of mutation—inherently less likely to occur [4]. One interpretation is that ‘earlier’ disruption in a more progenitor‐like/undifferentiated cell allows more rounds of division before postmitotic stalling, producing greater or longer lasting dysplasia. Consequently, a larger pool of premalignant cells is available to suffer a second hit and develop into cancer; therefore occurring statistically sooner. For the present, this remains speculation, although we note that methods to estimate the number of symmetric/asymmetric cell divisions prior to tumour initiation exist. For instance, by monitoring SNVs accumulating naturally through cell division coupled with multiregional tissue sampling and/or single‐cell sequencing [15] or by cell tracing experiments within mouse models [16]. Regardless, the answer surely lies in observing and/or modelling these putative PeRLs whose predetermined character may dictate an individual's tumour biology and outcome.

Critically, we note that two independent studies now confirm significant prognostic information, not readily apparent from DNA methylation subtyping, is encapsulated in a continuous manner within transcriptional patterns. This suggests a different approach to risk prognostication among MB_Grp3/Grp4_—as an alternative or adjunct to subcategorisation by subgroup—whereby a continuous base level of risk can be assigned to an individual patient based on their transcriptional profile, after which further independent risk modifiers (e.g., *MYC* amplification, presence of metastases, MB_Grp3/Grp4_ subgroup VII) may then be applied (Figure 1F).

The opportunities afforded by transcriptional analysis are perhaps best illustrated by those individuals who, by DNA methylation profiling, belong to MB_Grp3/Grp4_ subgroups II and III. These subgroups contain exclusively MB_Grp3_ individuals and, taken as a whole, demonstrate the classic poor prognosis historically associated with MB_Grp3_ [5]. Nevertheless, according to our transcriptional study [9], only ~40% of those patients could be considered to have a 5‐year survival <50% by virtue of their biology alone, that is, with no other risk modifiers taken into account. Similarly, Korshunov et al. [7] reported that the addition of *KIRREL2* expression to their biomarker scheme altered the risk category of up to 50% of MB_Grp3_ patients, with ~25% of such patients assigned to a low‐risk category with 5‐year OS 95%. Put simply, expression profiling can be deployed to reassign substantial proportions of patients more accurately within each MB_Grp3/Grp4_ subtype into ‘better’ or ‘worse’ treatment stratification groups. The job of upcoming clinical trials/biological studies will be to determine how best to deploy such information in the future.

We note that a surrogate G3/G4 continuum score can be derived from DNA methylation profiles—widely used for diagnosis—if no expression profile exists [9]. Conversely, a focussed diagnostic transcriptomic assay (qRT‐PCR, NanoString, etc.) could technically be used to achieve prognostication on its own, where no DNA methylation profile exists; however, we stress that we do not believe this to be desirable. We would expect progressive efforts in clinical trials and standard‐of‐care to continue, for the time being, to rely, in part, upon information more obtainable by DNA methylation analysis, and therefore, we imagine both expression and methylation assays operating side by side for the foreseeable future.

In short, there is now good evidence, backed up by a developmental/biological rationale, that expression is an effective prognostic determinant—particularly for MB_Grp3_. Further prospective clinical trials data will be required to definitively settle whether continuous or subgroup‐based prognostication, or a combination of both approaches, is most efficacious.

## AUTHOR CONTRIBUTIONS

All authors contributed to the conception and writing of the manuscript and approved the final version.

## CONFLICT OF INTEREST STATEMENT

The authors declare they have no conflict of interest.

## Data Availability

Data sharing is not applicable to this article as no new data were created or analyzed in this study.
